# Histopathological predictors of renal outcomes in idiopathic membranous nephropathy with a focus on global sclerotic glomeruli ratio

**DOI:** 10.1038/s41598-026-61455-0

**Published:** 2026-07-25

**Authors:** Onur Tunca, Aydin Turkmen, Necmi Eren, Serap Yadigar, Ferdi Karagoz, Mevlut Tamer Dincer, Mahmut Gok, Simal Koksal Cevher, Erhan Tatar, Sim Kutlay, Cebrail Karaca, Taner Basturk, Belda Dursun, Murat Duranay, Tuba Elif Ozler, Hakki Arikan, Kultigin Turkmen, Abdulmecit Yildiz, Rümeyza Kazancıoğlu, İsmail Kocyigit, Sena Ulu, Hamad Dheir, Arzu Özdemir, Sebnem Karakan, Alper Azak, Ebru Gok Oguz, Zülfükar Yılmaz, Garip Sahin, Mehmet Tanrisev, Erkan Sengul, Nedim Yılmaz Selcuk, Cuma Bulent GUL, Bulent Kaya, Melike Betul Ogutmen, Deren Oygar, Kenan Turgutalp, Hayriye Sayarlioglu, Ozkan Gungor, Mahmud Islam, Yagmur Tahillioglu, Sinan Kazan

**Affiliations:** 1 Faculty of Medicine, Department of Nephrology, Afyonkarahisar Health Sciences University, Afyonkarahisar, Turkey; 2 Faculty of Medicine, Department of Nephrology, Istanbul University, Istanbul, Turkey; 3 Faculty of Medicine, Department of Nephrology, Kocaeli University, Kocaeli, Turkey; 4 Clinic of Nephrology, Kartal Dr. Lutfi Kirdar City Hospital, Istanbul, Turkey; 5 Faculty of Medicine, Department of Nephrology, Trakya University, Edirne, Turkey; 6 Cerrahpasa Medical Faculty, Department of Nephrology, Istanbul University - Cerrahpasa, Istanbul, Turkey; 7 Department of Nephrology, University of Health Sciences, Abdülhamid Han Training and Research Hospital, Sultan 2, Istanbul, Turkey; 8 Ankara Bilkent City Hospital, Clinic of Nephrology, Ankara, Turkey; 9 Medical Point Hospital, Clinic of Nephrology, Izmir Economy University, Izmir, Turkey; 10 Faculty of Medicine, Department of Nephrology, Ankara University, Ankara, Turkey; 11 Faculty of Medicine, Department of Internal Medicine, Division of Nephrology, Van Yuzuncu Yil University, Van, Turkey; 12 Department of Nephrology, University of Health Sciences, Hamidiye Etfal Training and Research Hospital, Istanbul, Turkey; 13 Faculty of Medicine, Department of Nephrology, Pamukkale University, Denizli, Turkey; 14 Department of Nephrology, Ankara Training and Research Hospital, Ankara, Turkey; 15 Department of Internal Medicine, Division of Nephrology, Haseki Training and Research Hospital, Istanbul, Turkey; 16 Department of Nephrology, Marmara University School of Medicine, Istanbul, Turkey; 17 Faculty of Medicine, Department of Nephrology, Necmettin Erbakan University, Konya, Turkey; 18 Department of Nephrology, Bursa Uludag University School of Medicine, Bursa, Turkey; 19 Faculty of Medicine, Department of Nephrology, Bezmialem Vakif University, Istanbul, Turkey; 20 Department of Nephrology, Erciyes University Medical Faculty, Kayseri, Turkey; 21 Faculty of Medicine, Department of Nephrology, Sakarya University, Sakarya, Turkey; 22 Department of Nephrology, University of Health Sciences, Bakirkoy Dr. Sadi Konuk Training and Research Hospital, Istanbul, Turkey; 23 Faculty of Medicine, Department of Nephrology, Ankara Yildirim Beyazit University, Ankara, Turkey; 24 University of Health Sciences, Balikesir Ataturk Education and Research City Hospital, Balikesir, Turkey; 25 Department of Nephrology, University of Health Sciences, Etlik City Hospital, Ankara, Turkey; 26 Faculty of Medicine, Department of Nephrology, Dicle University, Diyarbakir, Turkey; 27 Faculty of Medicine, Department of Internal Medicine, Division of Nephrology, aa Eskisehir Osmangazi University, Eskisehir, Turkey; 28 Health Sciences University Izmir Tepecik Education and Research Hospital, Izmir, Turkey; 29 Hamidiye Faculty of Medicine, Department of Nephrology, ac University of Health Sciences, Kocaeli City Hospital, Kocaeli, Turkey; 30 Faculty of Medicine, Department of Nephrology, ad Cukurova University, Adana, Turkey; 31 Department of Nephrology, University of Health Sciences, Haydarpaşa Numune Training and Research Hospital, Istanbul, Turkey; 32 Division of Nephrology, af Dr Burhan Nalbantoglu State Hospital, Lefkosa, Cyprus; 33 Faculty of Medicine, Department of Internal Medicine, Division of Nephrology, ag Mersin University, Mersin, Turkey; 34 Faculty of Medicine, Department of Nephrology, Ondokuz Mayıs University, Samsun, Turkey; 35 Faculty of Medicine, Department of Nephrology, Kahramanmaras Sutcu Imam University, Kahramanmaras, Turkey; 36 Mavi Hastane , F Blok, Diyaliz Ünitesi, Merkez/Afyonkarahisar, Turkey

**Keywords:** Chronic kidney disease, glomerulosclerosis, global sclerotic glomeruli ratio, idiopathic membranous nephropathy, histopathology, prognostic marker, renal outcomes, tubular atrophy, Diseases, Medical research, Nephrology

## Abstract

Idiopathic membranous nephropathy (MN) shows variable renal outcomes, and reliable histologic markers for risk stratification are limited. The global sclerotic glomeruli ratio (GSGR) may reflect chronic kidney damage and help predict progression, but its prognostic value and optimal threshold remain unclear. We retrospectively analyzed 322 patients with biopsy-proven idiopathic MN from the Turkish Society of Nephrology-Glomerular Diseases Study Group registry, excluding those with an estimated glomerular filtration rate (eGFR) < 15 mL/min/1.73 m² at diagnosis or insufficient data. GSGR was calculated as the percentage of globally sclerotic glomeruli among total glomeruli. Histopathological parameters, including exudative glomerular changes, tubular atrophy/interstitial fibrosis (TA/IF), and vascular alterations, were semi-quantitatively graded according to the Renal Pathology Atlas (Fogo, 2022). Specifically, TA/IF was graded as: Grade 0 (< 5%), Grade 1 (5–25%), Grade 2 26–50%, and Grade 3 (> 50%) of cortical area involvement. Exudative changes were defined as intracapillary leukocyte accumulation and hyaline deposits in ≥ 25% of non-sclerotic glomeruli. The primary composite outcome was ≥ 50% eGFR decline from baseline, progression to eGFR < 15 mL/min/1.73 m², dialysis initiation, or kidney transplantation. Median follow-up was 60 months (IQR 48–72). Thirty patients (9.3%) reached the composite outcome. Receiver operating characteristic analysis identified a GSGR threshold of 15.7% (AUC 0.956; sensitivity 93.3%; specificity 82.7%). Patients with GSGR ≥ 15.7% had significantly higher event rates (35.1% vs. 1.2%; *p* < 0.001). In multivariate analysis, high GSGR, exudative changes, and response to immunosuppressive therapy independently predicted adverse outcomes. These associations remained significant across treatment subgroups. Sensitivity analyses confirmed these associations across multiple model specifications. Anti-PLA2R positivity correlated with higher baseline proteinuria but not independently with renal outcomes. A GSGR ≥ 15.7% is a strong, independent predictor of renal progression in idiopathic MN. Incorporating GSGR and exudative changes into risk stratification may improve prognostic assessment and therapeutic decision-making.

## Introduction

Chronic glomerular diseases are among the leading causes of end-stage renal disease (ESRD) worldwide, contributing significantly to the global burden of chronic kidney disease (CKD)^1^. In Turkey, registry data indicate that glomerular diseases rank as the fourth most common cause of ESRD, following diabetes mellitus, hypertension, and autosomal dominant polycystic kidney disease^2^. Glomerular diseases may manifest with distinct clinical syndromes, primarily nephrotic and nephritic syndromes. Among glomerular diseases, membranous nephropathy (MN) is the most frequent cause of nephrotic syndrome in adults^3,4^. MN can occur as a primary (idiopathic) form or secondary to malignancies, infections, medications, or systemic autoimmune diseases such as systemic lupus erythematosus^5^.

Idiopathic MN is now recognized as an autoimmune condition, typically associated with circulating autoantibodies against podocyte-specific antigens such as phospholipase A2 receptor (PLA2R) and thrombospondin type-1 domain-containing 7 A (THSD7A)^6^. While spontaneous remission occurs in approximately 30% of patients, those with persistent nephrotic proteinuria and no remission are at higher risk for ESRD. Several clinical and biochemical parameters—such as male sex, high proteinuria levels, elevated serum creatinine at diagnosis, and lack of remission—have been identified as poor prognostic indicators^7–9^.

Histopathological markers have gained attention as potential prognostic tools in glomerular diseases. The global sclerotic glomeruli ratio (GSGR), defined as the proportion of globally sclerotic glomeruli in a kidney biopsy specimen, is considered a histopathological marker of chronicity. Increasing evidence suggests that higher GSGR is associated with worse renal outcomes and greater risk of progression to ESRD^10–12^. However, an optimal cut-off value for GSGR in idiopathic MN remains undefined. Establishing such a threshold may enhance prognostic stratification in this population.

However, the relationship between GSGR and tubulointerstitial damage requires clarification. While tubular atrophy/interstitial fibrosis (TA/IF) has traditionally been considered the primary histological predictor of renal outcomes in MN [21–23], the independent contribution of GSGR relative to TA/IF remains uncertain. Furthermore, optimal quantification methods and thresholds for histopathological parameters, including exudative changes and TA/IF grading, have not been standardized in MN.

Therefore, this multicenter study aimed to^1^: evaluate the prognostic significance of GSGR in idiopathic MN and determine an optimal cut-off^2^; assess the relationship between GSGR and other pathological parameters, particularly TA/IF; and^3^ examine whether GSGR provides independent prognostic information beyond traditional chronicity indices.

## Results

### Study population

**Fig. 1:**
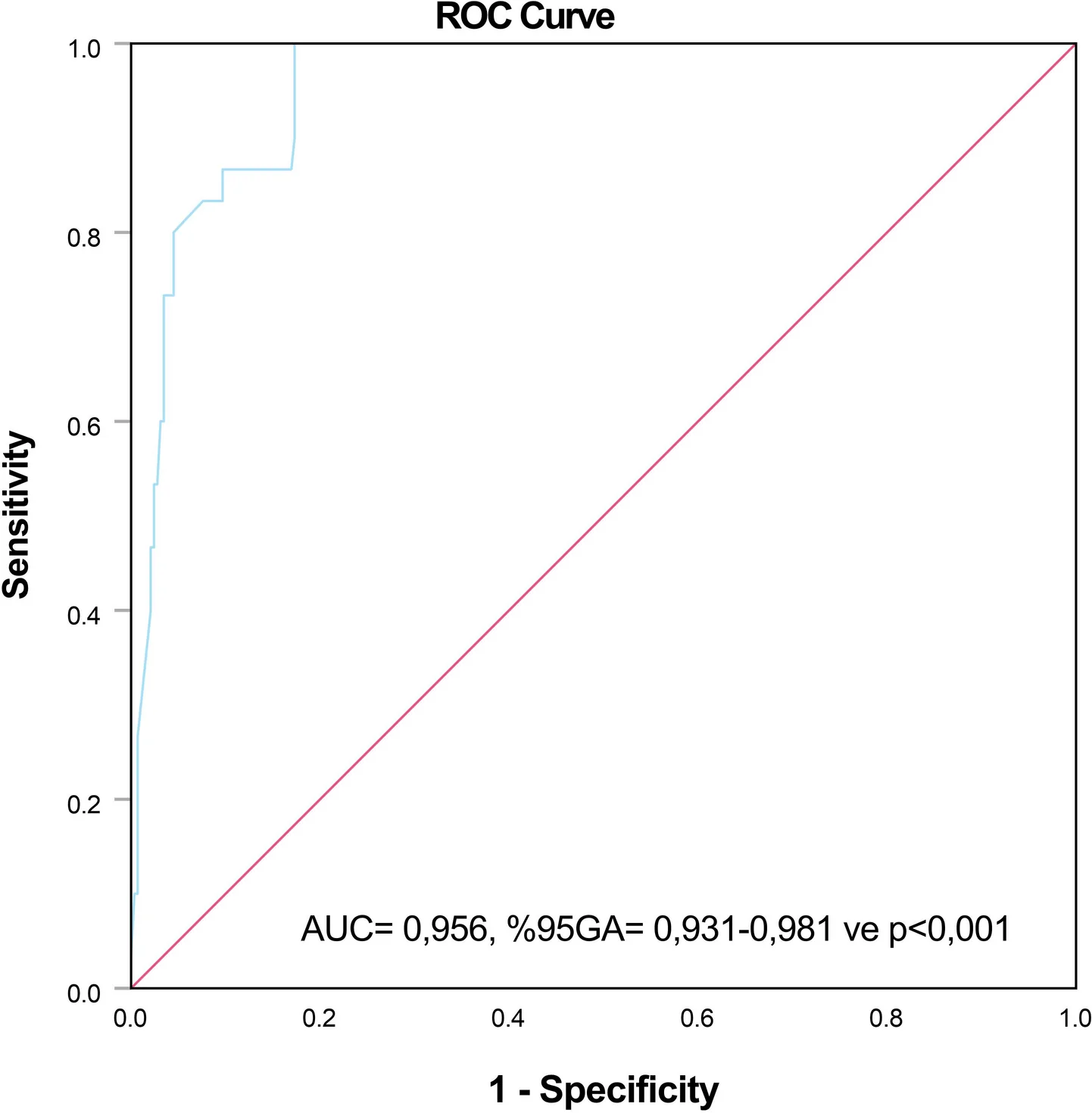
Flowchart illustrating the patient selection process. Out of 1,683 patients diagnosed with idiopathic membranous nephropathy (MN), 322 met the inclusion criteria for the final analysis after applying biopsy date, data sufficiency, and eGFR thresholds.

A total of 322 patients with biopsy-proven idiopathic membranous nephropathy (MN) were included in the final analysis (Fig. 1). The mean age at diagnosis was 44.6 ± 12.8 years, and 57.1% (*n* = 184) were male. Nephrotic syndrome was present in 87.3% (*n* = 281) of the cohort. Comorbid hypertension and diabetes mellitus were observed in 28.0% and 7.8% of patients, respectively, while 20.5% were active smokers. The median serum creatinine at diagnosis was within the normal range (patients with eGFR < 15 mL/min/1.73 m² were excluded).

The median follow-up duration was 60 months (interquartile range (IQR) 48–72 months), with no significant difference between those who reached the composite endpoint and those who did not (58 vs. 60 months, *p* = 0.670).

Histopathological staging classified patients as Stage 1 (*n* = 119, 37.0%), Stage 2 (*n* = 151, 46.9%), and Stage 3 (*n* = 52, 16.1%). Electron microscopy was available for only 15.2% (*n* = 49) of the patients. Baseline demographic, clinical, and pathological characteristics according to renal outcome status are presented in Table 1.

**Table 1:** Baseline Characteristics of the Study Population by Renal Outcome.

Variable	All Patients (*n* = 322)	Renal Endpoint (+) (*n* = 30)	Renal Endpoint (-) (*n* = 292)	*p*-value
Age at diagnosis, years (mean ± SD)	44.6 ± 12.8	45.3 ± 13.7	44.5 ± 12.7	0.659
Male sex, n (%)	184 (57.1%)	23 (76.7%)	161 (55.1%)	0.032
Hypertension, n (%)	90 (28.0%)	11 (36.7%)	79 (27.1%)	0.287
Diabetes mellitus, n (%)	25 (7.8%)	4 (13.3%)	21 (7.2%)	0.271
Active smoker, n (%)	66 (20.5%)	13 (43.3%)	53 (18.2%)	0.003
Nephrotic syndrome, n (%)	281 (87.3%)	29 (96.7%)	252 (86.3%)	0.149
Follow-up duration, months (median, IQR)	60 (48–72)	58 (46–71)	60 (48–72)	0.672
Immunosuppressive regimen, n (%)				0.421
- Cyclophosphamide-based	146 (45.3%)	12 (40.0%)	134 (45.9%)	
- Calcineurin inhibitor-based	92 (28.6%)	9 (30.0%)	83 (28.4%)	
- Rituximab-based	39 (12.1%)	5 (16.7%)	34 (11.6%)	
- Conservative only	45 (14.0%)	4 (13.3%)	41 (14.0%)	
Response to immunosuppressive therapy, n (%)	211 (65.5%)	12 (40%)	199 (68.2%)	< 0.001
Serum creatinine (mg/dL), median (IQR)	0.8 (0.6–1.1)	1.3 (0.9–1.73)	0.8 (0.6-1.0)	< 0.001
Serum albumin (g/dL), median (IQR)	2.7 (2.2–3.3)	2.3 (1.9–2.8)	2.7 (2.2–3.3)	0.001
Spot urine protein/creatinine ratio (mg/g), median (IQR)	5290 (4430–7440)	6850 (4430–8745)	4850 (4424–7319)	0.036
Tubular atrophy/IF grade, n (%)				0.018
- Grade 0 (< 5%)	196 (60.9%)	12 (40.0%)	184 (63.0%)	
- Grade 1 (5–25%)	87 (27.0%)	12 (40.0%)	75 (25.7%)	
- Grade 2 (26–50%)	28 (8.7%)	4 (13.3%)	24 (8.2%)	
- Grade 3 (> 50%)	11 (3.4%)	2 (6.7%)	9 (3.1%)	
Exudative glomerular changes, n (%)	39 (12.1%)	19 (63.3%)	20 (6.8%)	< 0.001
GSGR (%), median (IQR)	6.97 (0–16)	34.9 (26.6–41.6)	6.3 (0-11.8)	< 0.001
Electron microscopy available, n (%)	49 (15.2%)	5 (16.6%)	44 (15.1%)	0.642
Immunofluorescence findings:				
- IgG positive	322 (100%)	30 (100%)	292 (100%)	-
- C3 positive	288 (89.4%)	28 (93.3%)	260 (89.0%)	0.512
- IgA positive	40 (12.4%)	4 (13.3%)	36 (12.3%)	0.891
- IgM positive	76 (23.6%)	9 (30.0%)	67 (22.9%)	0.398
- Fibrinogen positive	27 (8.4%)	4 (13.3%)	23 (7.9%)	0.322
- C1q positive	20 (6.2%)	3 (10.0%)	17 (5.8%)	0.424

*GSGR* global sclerotic glomeruli ratio, *TA/IF* tubular atrophy/interstitial fibrosis.

### Clinical and pathological correlates

During a median follow-up of 5 years, 30 patients (9.3%) reached the composite renal endpoint. Among them, 15 (50%) experienced ≥ 50% eGFR decline, 7 (23.3%) progressed to eGFR < 15 mL/min/1.73 m², and 8 (26.7%) initiated renal replacement therapy.

The median global sclerotic glomeruli ratio (GSGR) for the cohort was 6.97% (IQR 0–16%). Patients who reached the composite endpoint had markedly higher GSGR values [median 34.9% (IQR 26.6–41.6%)] compared with those who did not [median 6.3% (IQR 0–11.8%)], *p* < 0.001. They also exhibited higher prevalence of exudative glomerular changes (63.3% vs. 6.8%, *p* < 0.001), tubular atrophy/interstitial fibrosis (60.0% vs. 37.0%, *p* = 0.018), and vascular alterations (53.3% vs. 32.9%, *p* = 0.024).

### ROC curve analysis and GSGR cut-off

**Fig. 2:**
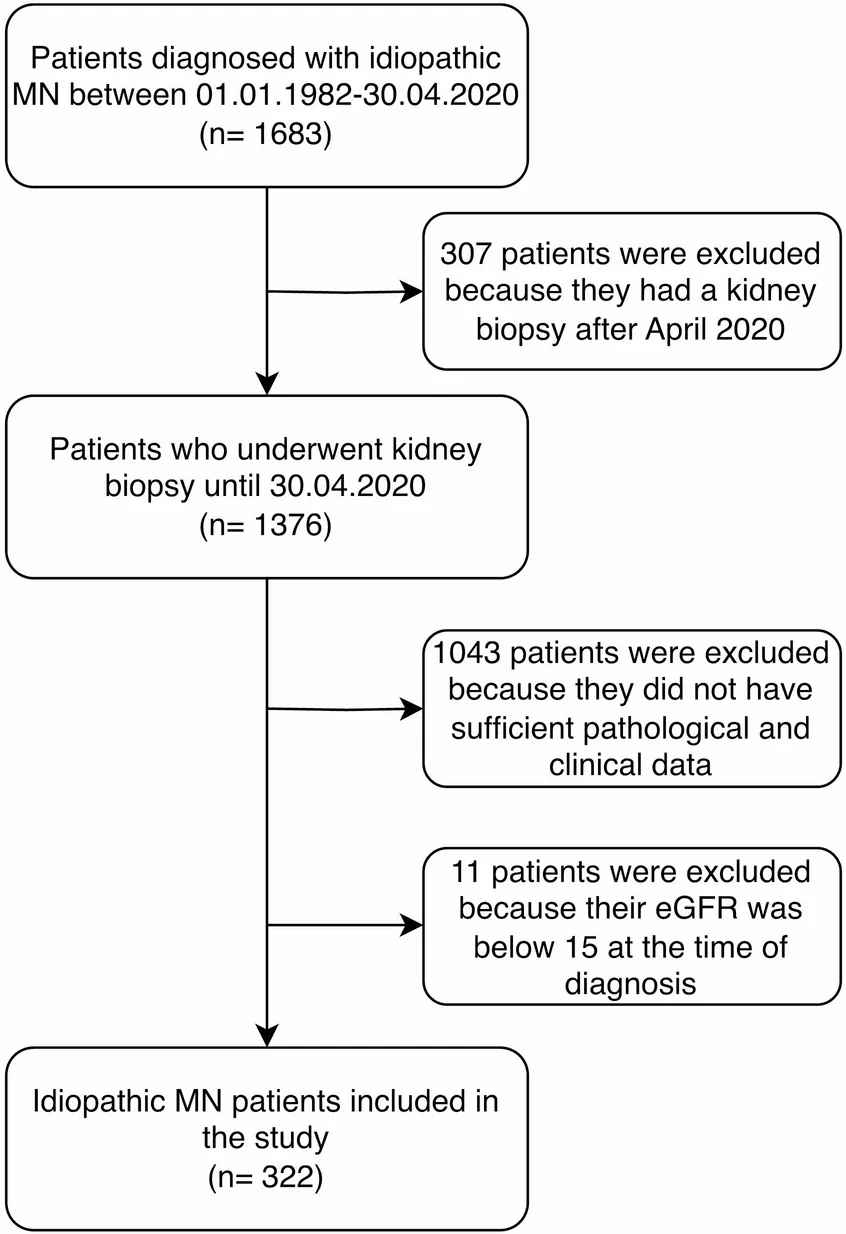
Receiver operating characteristic (ROC) curve for the global sclerotic glomeruli ratio (GSGR) predicting composite renal outcome. The area under the curve (AUC) was 0.956 (95% CI 0.931–0.981; *p* < 0.001), indicating excellent discriminative ability.

Receiver operating characteristic (ROC) curve analysis identified an optimal GSGR threshold of 15.7% for predicting the composite renal outcome, corresponding to an area under the curve (AUC) of 0.956 (95% CI 0.931–0.981, *p* < 0.001). This cut-off yielded 93.3% sensitivity and 82.7% specificity (Fig. 2). Based on this threshold, 77 patients (23.9%) were categorized as having high GSGR (≥ 15.7%), and 245 (76.1%) as low GSGR (< 15.7%).

Patients in the high-GSGR group exhibited higher rates of hypertension (41.6% vs. 23.7%, *p* = 0.003), more tubular atrophy/interstitial fibrosis (58.4% vs. 33.1%, *p* < 0.001), and more vascular changes (49.4% vs. 30.2%, *p* = 0.003). They also had higher baseline serum creatinine [1.1 mg/dL (IQR 0.7–1.5) vs. 0.8 mg/dL (IQR 0.6–1.0), *p* < 0.001], lower treatment response (75.9% vs. 88.8%, *p* = 0.018), and significantly higher incidence of renal events (35.1% vs. 1.2%, *p* < 0.001) (Table 2).

**Table 2:** Comparison of Clinical and Histopathological Features by GSGR Groups.

Variable	Low GSGR (< 15.7%) (*n* = 245)	High GSGR (≥ 15.7%) (*n* = 77)	*p*-value
Male sex, n (%)	134 (54.7%)	50 (64.9%)	0.146
Hypertension, n (%)	58 (23.7%)	32 (41.6%)	0.003
Active smoker, n (%)	44 (18.0%)	22 (28.6%)	0.052
Follow-up duration, months (median, IQR)	60 (48–72)	59 (47–71)	0.734
Response to immunosuppression, n (%)	167 (88.8%)	44 (75.9%)	0.018
Serum creatinine (mg/dL), median (IQR)	0.8 (0.6-1.0)	1.1 (0.7–1.5)	< 0.001
Tubular atrophy/IF, n (%)			< 0.001
- Grade 0	164 (67.0%)	32 (41.6%)	
- Grade 1	57 (23.3%)	30 (39.0%)	
- Grade 2	18 (7.3%)	10 (13.0%)	
- Grade 3	6 (2.4%)	5 (6.5%)	
Vascular changes, n (%)	74 (30.2%)	38 (49.4%)	0.003
Exudative glomerular changes, n (%)	19 (7.8%)	20 (26.0%)	< 0.001
Composite renal endpoint, n (%)	3 (1.2%)	27 (35.1%)	< 0.001

### Regression analyses

Univariate logistic regression identified several predictors of adverse renal outcomes (Table 3). In the multivariable model adjusted for age, serum creatinine (log-transformed), proteinuria, serum albumin, and TA/IF grade (0–3), three factors remained independently associated with renal progression:

**Table 3:** Risk factors of composite kidney outcome.

Characteristics	Univariate			Multivariate		
	OR	95% CI	*p*	OR	95% CI	*p*
Male gender (vs. female)	2.673	1.112–6.424	0.028	0.890	0.137–5.785	0.903
Active smoker (vs. non-smoker)	3.448	1.579–7.530	0.002	4.269	0.879–20.728	0.072
Therapeutic response (vs. no response)	0.151	0.059–0.385	< 0.001	0.128	0.020–0.801	0.028
Serum creatinine at diagnosis (per log-unit)	6.395	2.935–13.936	< 0.001	0.846	0.187–3.823	0.828
Serum albumin (per g/dL)	0.425	0.241–0.750	0.003	0.557	0.184–1.690	0.302
Spot urine protein/creatinine ratio (per 100 mg/g)	1.020	1.010–1.040	< 0.001	1.001	0.999–1.002	0.180
Exudative changes in the glomeruli (vs. absent)	23.491	9.838–56.093	< 0.001	43.131	7.151-260.156	< 0.001
Tubular atrophy/IF grade (per grade increase)	2.556	1.186–5.509	0.017	1.412	0.421–4.735	0.576
High GSGR (vs. low GSGR)	43.560	12.719-149.184	< 0.001	25.037	4.453-140.773	< 0.001

*GSGR* global sclerotic glomeruli ratio.

1. Therapeutic response (OR 0.128, 95% CI 0.020–0.801, *p* = 0.028).
2. Exudative glomerular changes (OR 43.131, 95% CI 7.151-260.156, *p* < 0.001).
3. High GSGR (OR 25.037, 95% CI 4.453-140.773, *p* < 0.001).

Model goodness-of-fit was confirmed by Hosmer-Lemeshow test (*p* = 0.780) and Nagelkerke R^2^= 0.412. Variance inflation factors (VIF) for all covariates were < 2.5, indicating no significant multicollinearity.

Sensitivity analyses using alternative model specifications (Table 4) confirmed the robustness of these associations.

**Table 4:** Sensitivity Analyses: Multivariate Models with Alternative Specifications.

Model Specification	High GSGR OR (95% CI)	*p*-value	Exudative Changes OR (95% CI)	*p*-value	TA/IF OR (95% CI)	*p*-value
Model 1: Original	25.037 (4.453-140.773)	< 0.001	43.131 (7.151-260.156)	< 0.001	1.412 (0.421–4.735)	0.576
Model 2: +Follow-up duration	24.832 (4.398-140.215)	< 0.001	42.891 (7.098-259.342)	< 0.001	1.398 (0.417–4.687)	0.589
Model 3: Excluding < 10 glomeruli	22.154 (3.821-128.442)	< 0.001	38.765 (6.234-240.982)	< 0.001	1.356 (0.398–4.621)	0.621
Model 4: TA/IF ordinal (0–3)	18.342 (3.214-104.523)	< 0.001	39.421 (6.542-237.812)	< 0.001	1.837 (1.124–3.021)	0.016
Model 5: Excluding eGFR < 30	28.421 (4.982-162.134)	< 0.001	45.982 (7.542-280.234)	< 0.001	1.298 (0.387–4.352)	0.678
Model 6: +Treatment regimen	23.987 (4.256-135.123)	< 0.001	41.234 (6.821-249.123)	< 0.001	1.378 (0.412–4.612)	0.602

TA/IF analyzed as ordinal variable (per grade increase) in Model 4; as binary (any vs. none) in other models. All models adjusted for age, serum creatinine (log−transformed), proteinuria, serum albumin, and TA/IF (except where noted).

When TA/IF was analyzed as a binary variable (presence vs. absence of any tubular damage), it was not independently associated with outcomes after adjustment for GSGR and other covariates (OR 1.412, 95% CI 0.421–4.735, *p* = 0.576). However, when analyzed as an ordinal variable with individual grades (0–3), each grade increment was significantly associated with adverse outcomes (OR 1.84 per grade increase, 95% CI 1.12–3.02, *p* = 0.016).

### Subgroup and correlation analyses

Subgroup analyses demonstrated consistent prognostic value of GSGR across treatment subgroups (Table 5):

**Table 5:** Subgroup Analyses: GSGR Prognostic Value Across Treatment Groups.

Treatment subgroup	*n*	High GSGR Event Rate	Low GSGR event rate	*p*-value
Conservative therapy only	45	40.0% (4/10)	0% (0/35)	0.003
Immunosuppressive responders	211	18.2% (8/44)	0.6% (1/167)	< 0.001
Immunosuppressive non-responders	66	61.8% (21/34)	12.5% (4/32)	0.008
Nephrotic syndrome present	281	38.2% (13/34)	1.4% (3/247)	< 0.001
Nephrotic syndrome absent	41	0% (0/6)	0% (0/35)	-

Event rate = proportion reaching composite renal endpoint. High GSGR defined as ≥15.7%.

- *Conservative therapy only* (*n* = 45): High GSGR associated with progression in 40.0% vs. 0% (*p* = 0.003).
- *Immunosuppressive therapy responders* (*n* = 211): High GSGR associated with progression in 18.2% vs. 0.6% (*p* < 0.001).
- *Immunosuppressive therapy non-responders* (*n* = 66): High GSGR associated with progression in 61.8% vs. 12.5% (*p* = 0.008).

In patients with nephrotic syndrome (*n* = 281), high GSGR was consistently associated with a higher incidence of renal endpoints. The prognostic value of high GSGR was also evident among treatment responders: even those who achieved immunosuppressive response and had high GSGR showed markedly greater progression risk compared to low-GSGR responders. Patients exhibiting both high GSGR and exudative changes (*n* = 20) showed the highest event rate (60%), while those without either feature (*n* = 183) rarely progressed (0.5%). Full data are presented in Table 5.

Correlation analysis revealed a moderate positive correlation between GSGR and TA/IF grade (Spearman’s ρ = 0.46, *p* < 0.001). Although these markers are related—both reflecting chronic kidney damage—the moderate rather than strong correlation indicates they are not collinear and likely capture different aspects of chronicity.

## Discussion

In this multicenter retrospective cohort study, we investigated the prognostic value of the global sclerotic glomeruli ratio (GSGR) in patients with idiopathic membranous nephropathy (MN). We identified a GSGR threshold of 15.7% that strongly predicted adverse renal outcomes with excellent discriminative accuracy (AUC 0.956). Patients with GSGR ≥ 15.7% were markedly more likely to reach the composite renal endpoint—defined as ≥ 50% eGFR decline, progression to eGFR < 15 mL/min/1.73 m², or initiation of renal replacement therapy.

Importantly, high GSGR, the presence of exudative glomerular changes, and response to immunosuppressive therapy independently predicted poor renal outcomes, even after adjustment for traditional clinical and biochemical risk factors.

Previous research has consistently identified tubulointerstitial damage as the primary histological predictor of renal outcomes in idiopathic MN. Studies by Wehrmann et al., Shiiki et al., and Stangou et al. all demonstrated that interstitial fibrosis and tubular atrophy are strongly associated with disease progression^13–15^. In most of these studies, global glomerulosclerosis was not independently predictive after adjustment for TA/IF.

Several factors may explain why GSGR emerged as the strongest predictor in our cohort:

First, our longer follow-up duration (median 60 months vs. typically 24–48 months in prior studies [21–23]) may have allowed chronic glomerular changes to manifest their full prognostic impact. We note, however, that follow-up durations in references 21–23 were broadly comparable to ours; therefore, this explanation may have limited force, and other differences in cohort characteristics or analytical approaches are likely contributing factors. Glomerulosclerosis may represent a slower, more insidious process that becomes apparent only with extended observation.

Second, our patient population had relatively preserved baseline renal function (eGFR ≥ 15 mL/min/1.73 m², median eGFR > 60 mL/min/1.73 m²). In cohorts with more advanced chronic kidney disease at presentation, TA/IF effects may dominate because tubulointerstitial damage is the final common pathway of all progressive renal diseases. In earlier disease stages, glomerular chronicity markers may have greater discriminative power.

Third, we used a quantitative GSGR ratio rather than categorical presence/absence of global sclerosis. While quantifying glomerulosclerosis as a percentage is standard practice, the use of a pre-specified threshold (15.7%) derived by ROC analysis—rather than relying on qualitative or arbitrary cutoffs—may have enhanced discrimination. This threshold-based approach allowed clearer stratification of patients into prognostically distinct groups.

Fourth, GSGR and TA/IF likely provide complementary rather than competing information. The moderate correlation (*r* = 0.46) between these markers suggests they capture different aspects of chronicity. Our finding that both remained independently predictive in multivariate models supports their additive prognostic value—GSGR reflecting chronic glomerular injury and TA/IF reflecting chronic tubulointerstitial damage.

The apparently high odds ratio for serum creatinine (OR 6.395 per log-unit) reflects the logarithmic transformation applied due to the skewed distribution of creatinine values. This translates to approximately 1.25-fold increased risk per 0.1 mg/dL increase in the normal range (e.g., 0.8 to 0.9 mg/dL), which is clinically plausible. Without transformation, extreme creatinine values would exert undue influence on the model.

Notably, Wei et al. similarly reported that glomerular sclerosis proportion ≥ 6.45% was associated with renal outcomes even after adjustment for tubular atrophy and interstitial inflammation in MN patients^16^.

This supports that our findings are not isolated and that global glomerulosclerosis may indeed have independent prognostic significance in specific MN populations.

Our findings highlight an important methodological consideration in the histopathological assessment of MN. Previous studies predominantly classified TA/IF as binary (present/absent), which may have obscured the true prognostic contribution of tubulointerstitial damage when adjusted for glomerular chronicity markers. The fact that ordinal TA/IF remained significant while binary TA/IF did not, after GSGR adjustment, suggests two possibilities: First, mild TA/IF (Grade 1, 5–25%) may not confer additional risk beyond glomerular damage in early disease stages. Second, the prognostic impact of TA/IF may follow a threshold effect, becoming apparent only at moderate-to-severe grades (≥ Grade 2). This aligns with the ‘chronicity threshold’ concept proposed by Fogo et al., where substantial interstitial fibrosis (> 25%) is required to independently influence renal trajectory. Our data support using ordinal TA/IF grading in future prognostic models, as binary classification may underestimate the contribution of tubulointerstitial damage.

The strong independent association between exudative glomerular changes and adverse outcomes (OR 43.1) warrants careful discussion. It is important to note that this association is based on the operational definition used in this study—defined as intracapillary leukocyte accumulation and hyaline deposits occluding the glomerular lumen in ≥ 25% of non-sclerotic glomeruli—which encompasses more than one histopathological component. Given this composite nature, mechanistic interpretations should remain cautious and should not be extrapolated beyond this specific definition. With this caveat in mind, the prognostic association may reflect several overlapping processes:

1. *Capillary wall stress*: Severe proteinuria and glomerular hypertension may cause endothelial dysfunction and protein leakage into the capillary lumen, forming hyaline deposits.
2. *Complement-mediated injury*: In PLA2R-associated MN, complement activation may cause endothelial injury and inflammatory cell recruitment.
3. *Superimposed pathology*: Prominent exudative changes should raise consideration of secondary MN (infection-related, malignancy-associated) or superimposed thrombotic microangiopathy, though we excluded known secondary causes clinically.

Taken together, these features may identify a high-risk MN phenotype with active glomerular injury beyond the typical subepithelial immune complex deposition. However, given that the variable is operationally defined and encompasses heterogeneous histopathological elements, the underlying biological mechanisms remain to be clarified in future studies with more granular pathological characterization.

### Clinical implications

Our findings suggest that integrating quantitative histopathological features—specifically GSGR and exudative changes—into clinical risk models can refine prognostic stratification in idiopathic MN. GSGR can be easily calculated from standard biopsy reports without additional cost. Including this parameter alongside clinical markers (proteinuria, eGFR) and serologic markers (anti-PLA2R) may help identify patients requiring closer monitoring or earlier therapeutic intervention.

### Limitations

This study has several limitations. First, its retrospective design may introduce inherent biases. Second, while biopsies were centrally re-evaluated by two blinded pathologists (κ = 0.82), interobserver variability cannot be entirely excluded. Third, serum creatinine was used as a covariate rather than eGFR; this was because eGFR-derived values were not uniformly available across all centers in the registry, while serum creatinine was consistently recorded. We acknowledge that eGFR would be preferable as it adjusts for age and sex—factors that independently influence creatinine levels—and this represents a limitation of the study. Fourth, the TSN-GOLD registry captures endpoint occurrence but not precise event dates; therefore, we analyzed endpoint occurrence (yes/no) during the follow-up period rather than time-to-event. Follow-up duration was calculated from biopsy date to last available visit or endpoint occurrence, and we included follow-up duration as a covariate in all regression models. Prospective studies with complete event-date documentation should employ Cox proportional hazards regression.

Fifth, the relatively small number of events (*n* = 30) requires cautious interpretation. We addressed potential overfitting through sensitivity analyses with varying covariate sets (Table 4). Moreover, the events-per-variable (EPV) ratio of 3.3 (30 events / 9 variables) is below the conventional threshold of 10; readers should therefore interpret multivariable results with caution regarding potential model overfitting, biased odds ratios, unreliable 95% confidence intervals, and limited generalisability. In particular, the magnitude of the multivariable effect estimates for high GSGR (OR 25.037, 95% CI 4.453–140.773) and exudative glomerular changes (OR 43.131, 95% CI 7.151–260.156) should be interpreted with considerable caution: the wide confidence intervals reflect the limited number of events and indicate substantial statistical uncertainty around these point estimates. These findings are therefore best regarded as hypothesis-generating rather than definitive effect size estimates. A propensity score approach could serve as an alternative adjustment strategy in this low-EPV context, but was not applied in the current analysis. Sixth, heterogeneity in treatment protocols among centers could affect outcome comparability; however, subgroup analyses across treatment groups demonstrated consistent GSGR effects (Table 5).

Seventh, the GSGR threshold of 15.7% was derived and evaluated within the same cohort using ROC analysis, without internal cross-validation or external validation in an independent dataset. This represents an important limitation: thresholds derived in this manner are prone to optimism and may not retain their discriminative performance in different populations or clinical settings. Although the threshold appears clinically promising, it should not be applied broadly until it has been validated in independent prospective cohorts. External validation will be a critical step before this cut-off can be recommended for routine clinical use.

## Conclusions

A global sclerotic glomeruli ratio (GSGR) ≥ 15.7% is a strong, independent predictor of adverse renal outcomes in idiopathic membranous nephropathy, providing prognostic information additive to traditional chronicity indices such as tubular atrophy/interstitial fibrosis. The prognostic significance of GSGR persists across treatment subgroups and is independent of treatment response. Exudative glomerular changes represent an additional high-risk histological feature. Integrating these quantitative pathological parameters into routine biopsy reporting may enhance risk assessment and guide therapeutic decision-making. Future prospective studies should validate these findings and explore optimal integration of histological and clinical risk markers. Given the exploratory nature of the present analysis—particularly in light of the low EPV ratio and registry-based design—these results should be considered hypothesis-generating and require confirmation in larger prospective cohorts before informing clinical practice.

## Methods

### Study design

This multicenter retrospective cohort study was conducted using data from the Turkish Society of Nephrology–Primary Glomerular Diseases (TSN-GOLD) registry, a nationwide database comprising biopsy-proven primary glomerular diseases. Patients with a histopathological diagnosis of idiopathic membranous nephropathy (MN) were identified and their clinical and pathological data retrospectively reviewed. To ensure at least five years of follow-up, patients who underwent kidney biopsy after April 2020 were excluded.

### Patient selection

Inclusion criteria were^1^: biopsy-proven idiopathic MN^2^; baseline estimated glomerular filtration rate (eGFR) ≥ 15 mL/min/1.73 m²; and^3^ available clinical and laboratory follow-up data for at least five years. Secondary causes of MN (autoimmune diseases, infections, malignancies, or drug-induced cases) were excluded based on clinical and serologic evaluation at the time of diagnosis. After exclusions, 322 patients fulfilled the eligibility criteria and were included in the final analysis (Fig. 1).

### Data collection

Baseline demographic variables (age, sex), comorbidities (hypertension, diabetes mellitus, smoking status), and clinical presentation (presence of nephrotic syndrome) were recorded. Laboratory data included serum creatinine, albumin, hemoglobin, blood urea nitrogen (BUN), and spot urine protein-to-creatinine ratio. Histopathological data encompassed total and globally sclerotic glomeruli counts, exudative glomerular changes, tubular atrophy/interstitial fibrosis (TA/IF), vascular alterations, and presence of subepithelial deposits.

Anti-PLA2R antibody testing was available in 78% of patients after 2016 and THSD7A testing in 34% of PLA2R-negative cases. PLA2R positivity correlated with higher baseline proteinuria but not independently with renal outcomes.

Treatment variables (immunosuppressive regimen and treatment response) were documented. Response was defined as ≥ 50% reduction in proteinuria with stable or improved eGFR at 6 months.

### Histopathological assessment and GSGR calculation

Kidney biopsies were processed using standard light microscopy, immunofluorescence (IF), and electron microscopy (EM) when available (15.2% of cases). IF analyses included IgG, IgA, and C3 staining patterns, while EM confirmed the presence of subepithelial deposits.

The global sclerotic glomeruli ratio (GSGR) was calculated as the number of globally sclerotic glomeruli divided by the total number of glomeruli, expressed as a percentage.

Exudative glomerular changes, tubular atrophy/interstitial fibrosis, and vascular alterations were graded semi-quantitatively according to the Renal Pathology Atlas (Agnes Fogo, 2022):

- *Tubular atrophy/interstitial fibrosis (TA/IF):* Grade 0 = none (< 5% of cortical area); Grade 1 = mild (5–25%); Grade 2 = moderate (26–50%); Grade 3 = severe (> 50%). For regression analyses, “tubular damage” was defined as Grade ≥ 1 (≥ 5% involvement).
- *Exudative changes:* Defined as intracapillary leukocyte accumulation and hyaline deposits occluding the glomerular lumen in ≥ 25% of non-sclerotic glomeruli in the biopsy specimen. We acknowledge that this operational definition encompasses both inflammatory features (endocapillary hypercellularity with neutrophils) and chronic/sclerosing features (hyalinosis), which differ from the consensus KI 2020 definitions of “exudative lesions” and “hyalinosis” as distinct entities. This composite definition was chosen to capture the overall severity of intracapillary occlusive lesions in our cohort, and readers should interpret our findings in light of this operational classification.
- *Vascular changes:* Defined as intimal thickening exceeding media thickness or arteriolar hyalinosis affecting ≥ 25% of vessels examined.

**Fig. 3:**
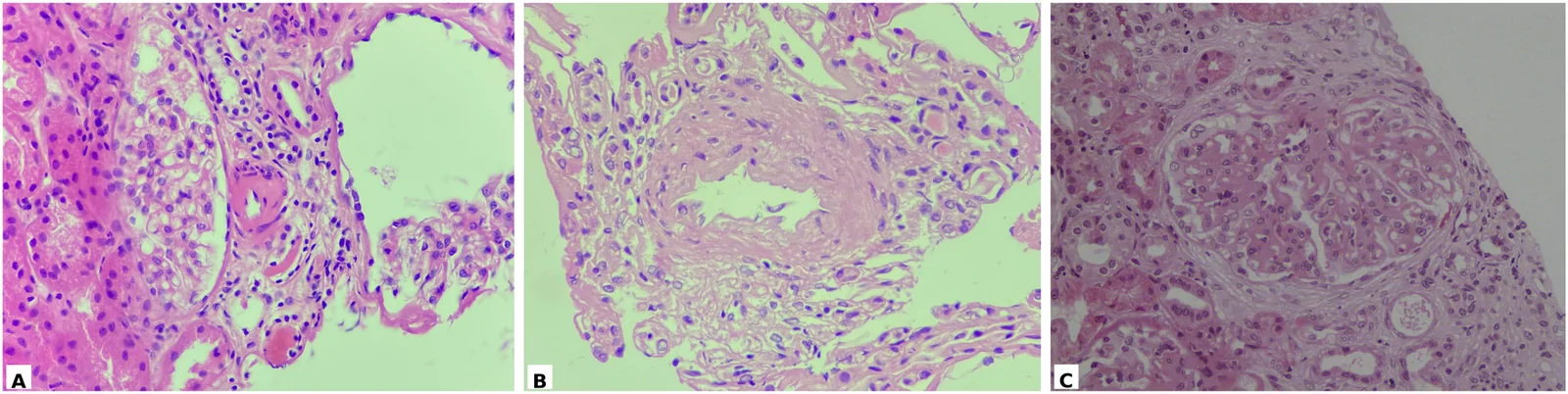
Representative histopathological images. (**A**) Hypertrophy of the tunica media in the arterial wall (H&E stain, ×400). (**B**) Arteriolar hyalinization (H&E stain, ×400). (**C**) A glomerulus showing diffuse basement membrane thickening and occasional intracapillary leukocyte infiltration (H&E stain, ×400).

**Fig. 4:**
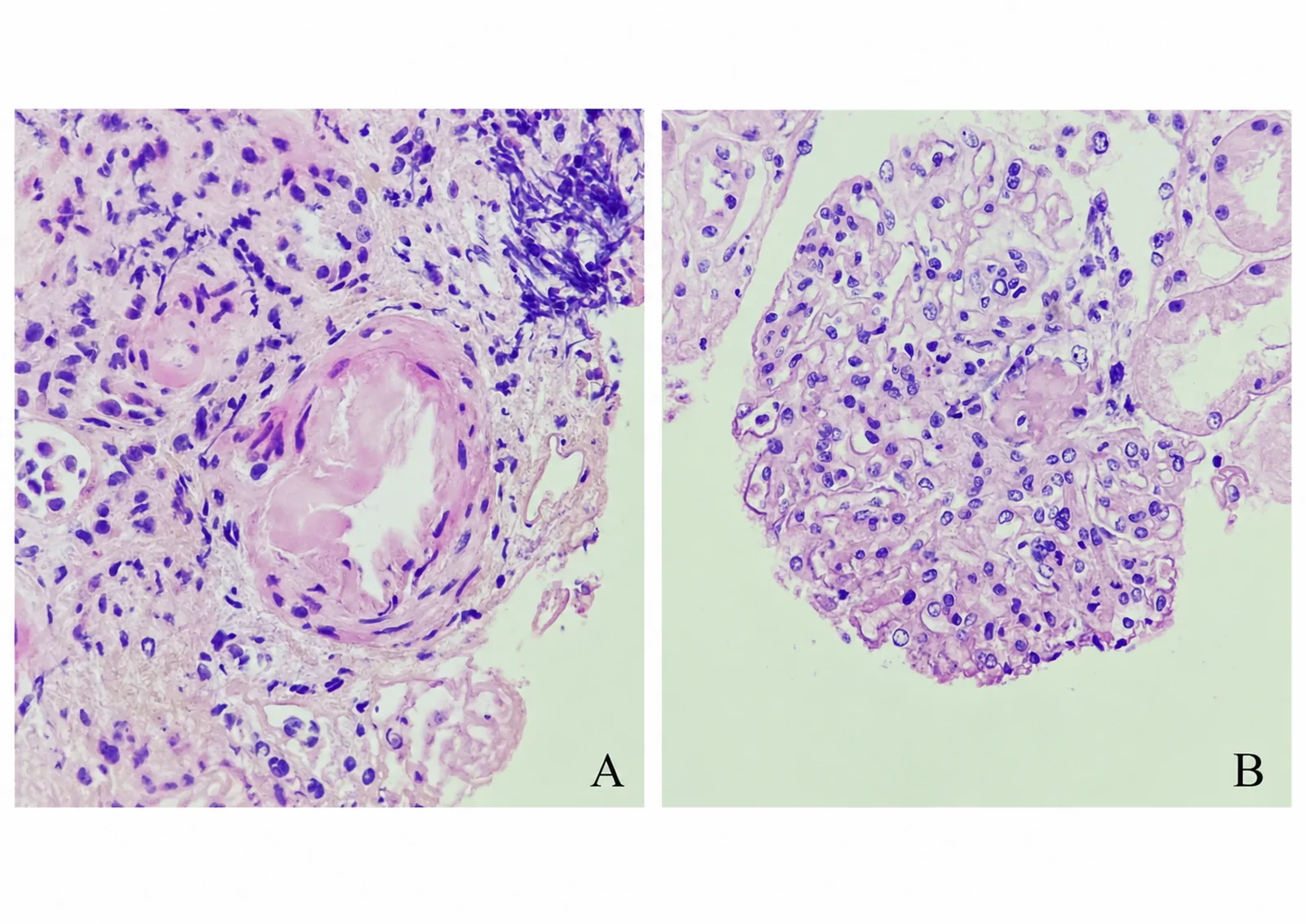
Representative histopathological images. (**A**) Arteriolar hyalinosis (H&E stain, ×400). (**B**) Polymorphonuclear leukocytes within the vascular lumina and mesangial matrix accumulation (H&E ×400).

Representative histopathological images are shown in Figs. 3 and 4.

All biopsies were re-evaluated centrally by two renal pathologists blinded to clinical data, achieving an interobserver κ = 0.82.

Biopsy adequacy was defined as ≥ 8 glomeruli per specimen. Patients with < 10 glomeruli (*n* = 31, 9.6%) were included in primary analyses but excluded in sensitivity analyses.

### Histopathological staging

Patients were classified into histopathological stages 1–3 based on glomerular morphology, as recommended in current MN classifications. Stage 1: minimal thickening with small subepithelial deposits; Stage 2: moderate thickening with spike formation; Stage 3: advanced thickening with basement-membrane projections and global sclerosis. The distribution of stages is presented in Table 1.

### Definition of renal outcomes

The composite renal endpoint was defined as the occurrence of any of the following during follow-up^1^:≥ 50% decline in eGFR from baseline^2^; progression to eGFR < 15 mL/min/1.73 m²^3^; initiation of renal replacement therapy (hemodialysis, peritoneal dialysis, or kidney transplantation). Patients were categorized as “endpoint +” or “endpoint -” according to this definition. The TSN-GOLD registry captures endpoint occurrence but not precise event dates; therefore, we analyzed endpoint occurrence (yes/no) during the follow-up period rather than time-to-event. Follow-up duration was calculated from biopsy date to last available visit or endpoint occurrence.

### Statistical analysis

All analyses were performed using SPSS version 27.0.1 (IBM Corp., Armonk, NY, USA). Continuous variables were assessed for normality using the Shapiro–Wilk test and visual histogram inspection. Normally distributed variables were expressed as mean ± standard deviation (SD), and non-normally distributed variables as median (interquartile range, IQR).

Categorical variables were summarized as frequencies and percentages. Between-group comparisons used the chi-square or Fisher’s exact test for categorical data, independent-samples t-test for normally distributed variables, and Mann–Whitney U test for non-parametric data.

Receiver operating characteristic (ROC) curve analysis determined the optimal GSGR threshold for predicting the composite endpoint, using the Youden index.

*Correlation between GSGR and TA/IF grade was assessed using Spearman’s rank correlation coefficient (ρ).* “TA/IF was analyzed both as a binary variable (any damage [Grade ≥ 1] vs. none [Grade 0]) and as an ordinal variable (Grades 0–3). The ordinal approach was preferred a priori as it preserves the graded nature of tubulointerstitial damage. However, we present both approaches to facilitate comparison with prior studies that predominantly used binary classifications.”

Due to the right-skewed distribution of serum creatinine and the non-linear relationship between creatinine levels and renal outcomes, serum creatinine was natural log-transformed for regression analyses. Results are presented as odds ratios per 1-unit increase in log-transformed creatinine (approximately equivalent to 2.72-fold increase in raw creatinine values). For clinical interpretability, we also calculated the OR per 0.1 mg/dL increase in raw creatinine values (OR: 1.21, 95% CI 1.08–1.36, *p* < 0.001).

Variables showing significant associations in univariate analyses were entered into multivariable logistic regression using the enter method. Covariates included age, serum creatinine (log-transformed, as a proxy for renal function given the absence of uniformly available eGFR values across centers), proteinuria, serum albumin, TA/IF grade (0–3), and follow-up duration.

Binary logistic regression was used for endpoint analysis rather than Cox proportional hazards regression. Although the TSN-GOLD registry records follow-up duration in months, it does not capture the precise date at which each endpoint occurred during follow-up. Using total follow-up duration as a surrogate for time-to-event in a Cox model would implicitly assume that the event occurred at the end of the observation period for all patients, introducing systematic bias in hazard estimates. Therefore, endpoint occurrence (yes/no) was analyzed as a binary outcome, with follow-up duration included as a covariate in all models.

Model goodness-of-fit was assessed using the Hosmer-Lemeshow test and Nagelkerke R². Multicollinearity was evaluated using variance inflation factors (VIF); VIF > 2.5 was considered indicative of significant multicollinearity. Standardized residuals and Cook’s distance were examined to identify influential outliers.

Sensitivity analyses were performed to assess result robustness:

1. Excluding patients with < 10 glomeruli (*n* = 31).
2. Excluding patients with serum creatinine corresponding to eGFR < 30 mL/min/1.73 m² (*n* = 28).
3. Using TA/IF as ordinal variable (grades 0–3) rather than binary.
4. Adjusting for immunosuppressive regimen type.
5. Adjusting for follow-up duration as additional covariate.

A two-tailed *p* < 0.05 was considered statistically significant.

## Data Availability

The dataset analyzed during the current study is part of the Turkish Society of Nephrology Primary Glomerular Diseases Database. This database is not publicly available. Access is restricted and requires permission from the Turkish Society of Nephrology. Data can be obtained from the society upon reasonable request and with approval of the governing board.
